# Energy determines broad pattern of plant distribution in Western Himalaya

**DOI:** 10.1002/ece3.3569

**Published:** 2017-11-10

**Authors:** Rajendra M. Panda, Mukunda Dev Behera, Partha S. Roy, Chandrashekhar Biradar

**Affiliations:** ^1^ School of Water Resources Indian Institute of Technology Kharagpur West Bengal India; ^2^ Centre for Oceans, Rivers, Atmosphere and Land Sciences (CORAL) Indian Institute of Technology Kharagpur West Bengal India; ^3^ Centre for Earth and Space Sciences University of Hyderabad Hyderabad India; ^4^ International Center for Agricultural Research in Dry Areas (ICARDA) CGIAR Amman Jordan

**Keywords:** climate, generalized additive model, species richness, structural equation model, water–energy dynamics

## Abstract

Several factors describe the broad pattern of diversity in plant species distribution. We explore these determinants of species richness in Western Himalayas using high‐resolution species data available for the area to energy, water, physiography and anthropogenic disturbance. The floral data involves 1279 species from 1178 spatial locations and 738 sample plots of a national database. We evaluated their correlation with 8‐environmental variables, selected on the basis of correlation coefficients and principal component loadings, using both linear (structural equation model) and nonlinear (generalised additive model) techniques. There were 645 genera and 176 families including 815 herbs, 213 shrubs, 190 trees, and 61 lianas. The nonlinear model explained the maximum deviance of 67.4% and showed the dominant contribution of climate on species richness with a 59% share. Energy variables (potential evapotranspiration and temperature seasonality) explained the deviance better than did water variables (aridity index and precipitation of the driest quarter). Temperature seasonality had the maximum impact on the species richness. The structural equation model confirmed the results of the nonlinear model but less efficiently. The mutual influences of the climatic variables were found to affect the predictions of the model significantly. To our knowledge, the 67.4% deviance found in the species richness pattern is one of the highest values reported in mountain studies. Broadly, climate described by water–energy dynamics provides the best explanation for the species richness pattern. Both modeling approaches supported the same conclusion that energy is the best predictor of species richness. The dry and cold conditions of the region account for the dominant contribution of energy on species richness.

## 1 INTRODUCTION

Several factors describe the broad pattern of diversity in plant species distribution of which climate is a critical factor (Currie, 2004) at a regional scale (Ricklefs, 1987) and in tropical forests (Clark et al., 1999). The water–energy dynamics hypothesis explains the mechanism of climatic control and the positive relationships that ambient energy availability and water have with species richness (O'Brien, 1993). The energy hypothesis explains energy partitioning among species where greater availability of energy in a usable form supports more species (Turner, Gatehouse, & Corey, 1987). In general, there is a proven high positive correlation between energy and species richness in cold climates. However, there are variations in these relationships in warm conditions (Francis & Currie, 2003; Kreft & Jetz, 2007). Water is an essential solvent for all physiological activities in plants and determines species patterns in the tropics, subtropics, and warm temperate zones (Hawkins et al., 2003). The scarcity of water adversely affects plant species richness in arid climates (Li et al., 2013). Water, energy, and their dynamics determine plant richness at high latitudes and explain more than 60% of the variations found in plant/animal species richness (Hawkins et al., 2003). However, the individual contributions of water and energy vary within regions. Vetaas and FerrerCastán (2008) observed that energy exerts greater control than does precipitation over the woody plants of the Iberian Peninsula. In contrast, Hawkins, Diniz‐Filho, Jaramillo, and Soeller (2006) demonstrated a stronger influence of precipitation relative to energy in high to mid‐latitudes.

Physiography describes the geographic complexity and regulates species diversity at both local and regional scales (Moeslund, Arge, Bøcher, Dalgaard, & Svenning, 2013). Several physiographic variables are used to quantify its impacts on species richness. Elevation regulates species richness by controlling the effects of climate and soil (Day & Monk, 1974), and it strongly influences the vegetation of most mountain ecosystems (Zhang et al., 2009). The terrain ruggedness index obtained by the differences between the elevation values of adjacent cells relative to a central cell (Riley, Hoppa, Greenberg, Tufts, & Geissler, 2000) describes the impacts of heterogeneity in species’ niche differentiation (Whittaker, Levin, & Root, 1973). Aspect distributes the solar radiation affecting the microclimate and vegetation at a local scale (Kirkby et al., 1990). Slope correlates with the spatial pattern of tree species by controlling solar radiation (Bianchini, Garcia, Pimenta, & Torezan, 2010).

Disturbance is a critical factor for community composition and diversity in the species‐rich landscapes of Western Himalaya (Kharkwal, 2009). Roy et al. (2002) used patch density, porosity, fragmentation, and juxtaposition to assess the disturbance regime of the region. Sharma, Gairola, Ghildiyal, and Suyal (2009) found a positive correlation between poor socio‐ecological status of villagers and fuelwood collection in the temperate forests of the Garhwal Himalaya. Overgrazing is reported to be another factor in the alpine grasslands of the Tibetan plateau (Yu et al., 2012). Both the human appropriations of net primary productivity and the global human footprint index, which assess the intensity of human intervention in ecosystems and its sustainability, have been used as socio‐ecological indicators. Crowther et al. (2015) studied the effects of these variables to quantify human interference when mapping tree density at a global scale. Here, we emphasize their influence to monitor impacts of human disturbance on species richness. This has never been attempted previously in Western Himalayan studies.

Western Himalaya is geomorphologically complex with an altitudinal extent of >8,000 m. Its rich species diversity is broadly characterized by physiography, climate, soil, and anthropogenic disturbance (Shah et al., 2011). Its biogeography is climatologically distinct. It lies in a rain shadow region and receives little rainfall (Singh & Singh, 1987). Western Himalaya gets rainfall during winter due to nonmonsoonal precipitation of westerlies. Overall, a temperate climate prevails. However, the region experiences a broad range of temperature and precipitation anomalies with a mean annual temperature of *ca*. 5°C and annual precipitation of 2,500 mm (Chitale, 2014). The southern parts of the region are species‐rich. For example, the angiosperm diversity of Himachal Pradesh comprises about 19,395 species or 7% of the world total (Karthikeyan, 2000). In contrast, the northern part is species poor due to the very low rainfall it receives (10–70 mm) and extremely cold temperature (≤45°C). The published literature focuses on the species diversity, community structure, and distribution pattern of its different forest types (Khan, Page, Hahmad, & Harper, 2013; Shaheen, Khan, Harper, Ullah, & Allem Qureshi, 2012; Sharma, Rana, Devi, Randhawa, & Kumar, 2014; Singh, 2008). Some studies have been carried out on species–environment relationships in adjacent areas (Oommen & Shanker, 2005; Wang, Tang, & Fang, 2007; Yan, Yang, & Tang, 2013). However, no specific study has been performed in Western Himalaya due to nonavailability of floral data. In this study, we took advantage of a scientifically designed national database to investigate the influence of the abiotic environment on species richness. Understanding high species richness in a dry and cold climate of the understudied Himalaya remains interesting, and little research has focused on disentangling the effects of abiotic and human impacts. This study may provide better insights into ecologists and planners dealing with plant distribution patterns vis‐à‐vis climate change projections.

In this study, we explored the determinants of species richness in Western Himalayas. We used high‐resolution species data available for the area to energy, water, physiography, and anthropogenic disturbance to explain distribution of species richness. Because of geomorphological complexity, we expected a greater control of physiography on the species richness. The general dry and cold conditions of the area may posit significant impacts of water and energy on species distribution. We speculate anthropogenic disturbance has some control on the plant richness because of the proximity of the region to densely populated areas. We applied predictive models to assess the influences of environmental variables and human impact on the species richness.

## 2 METHODS

**Figure 1:**
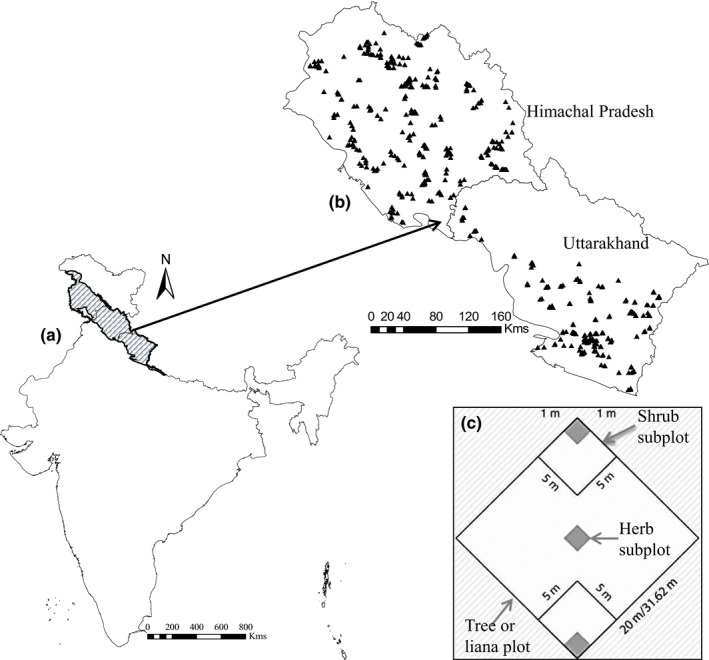
(a) Indian Parts of Western Himalaya. (b) Study area showing two Indian states with nested quadrat locations. (c) Design of a nested quadrat

We selected two Indian Himalayan states, Himachal Pradesh and Uttarakhand (28.5–33°N and 75.5–82°E), for the study (Figure 1). We obtained plant species richness data from a national database developed during the project “Biodiversity Characterization at Landscape Level” (bis.iirs.gov.in). The field sampling involving all the forest classes was carried out using a stratified random sampling approach for the vascular plant species richness (Roy et al., 2012). A nested quadrat of 20 × 20 m was laid for trees or lianas, which accommodated two 5 × 5 m quadrats for shrubs/saplings and five 1 × 1 m quadrats for herbs/seedlings (Figure 1). We gathered data pertaining to 27 variables under three categories, viz. climate (21 variables), physiography (four variables), and human disturbance (two variables) from different public domain sources (Appendix S1). We obtained 19 climate variables from the Worldclim site, along with two variables from CGIAR_CSI (http://www.cgiar-csi.org/), all computed for the period from 1950 to 2000 (Hijmans, Cameron, Parra, Jones, & Jarvis, 2005; Zomer, Antonio, Deborah, & Louis, 2008). We obtained physiographic variables (elevation, slope, and aspect) from the GMTED2010 database (https://lta.cr.usgs.gov). We derived the terrain ruggedness index from the differences of the elevation values of adjacent cells relative to a central cell. We procured the human appropriation of net primary productivity (Imhoff et al., 2004) and global human footprint (WCS‐CIESIN, 2005) values from the SEDAC database (http://sedac.ciesin.columbia.edu/). The spatial resolution of each of the 27 variables was approximately 1 km^2^. The data conformed to WGS'84 projection. We extracted the data corresponding to 1,178 species locations using ArcGIS 10 for regression analysis (see Appendix S1).

Spatial autocorrelation describes the dependencies between observed samples and bias due to clustering, which can be quantified using Moran's I index (Moran, 1950). Principal component analysis (PCA) was used to select the best among correlated predictors (Xu, Wang, Rahbek, Sanders, & Fang, 2016). We computed Moran's I index of the floral data using the package “ape” in RStudio (Paradis, Claude, & Strimmer, 2004). We performed a multicollinearity test through hierarchal clustering analysis using the package “corrplot” in RStudio (Wei & Simko, 2016). We used PCA to examine the loadings of each collinear variable of the different categories separately and to pick a few least‐correlated variables from each category for further analysis. We performed simultaneous centering and scaling to nullify the skewness effect. We performed PCA using the package “caret” in RStudio (Abdi & Williams, 2010). We calculated the percentage absolute weight of each variable corresponding to each principal component (PC) axis by multiplying the percentage variance explained by each axis with the absolute weight of each variable corresponding to that PC axis. We added the values of the first three PC axes, picked up the variable with the maximum weight, and removed its collinear partners (*R* > 0.8). We continued this process and finally selected the top eight variables. These include four climate (two energy and two water variables), two physiography and two disturbance variables. As two disturbance variables were noncollinear, we selected them directly without PCA. We examined the multicollinearity between the selected variables further to cross‐check whether they satisfied correlation criteria (*R* < 0.8).

Several species distribution models (SDMs) deal with linear or nonlinear species–environment relationships. We selected the generalized additive model (GAM) for its robustness and proficiency in predicting species richness pattern (Hastie & Tibshirani, 1990). GAM fits smooth functions to establish nonlinear relations between species and the environment (Vetaas & FerrerCastán, 2008). The cubic spline smoother reduces the curvature and straightens curves fitted with large values and vice versa. The tensor product smoothing function scales anisotropic parameters of different units and improves the performance of models with interactive terms. GAM provides sufficient flexibility to choose smoothing parameters and is capable of fitting several prediction error criteria to control overfitting. Generalized cross‐validation (GCV) and generalized (approximate) cross‐validation (GACV) are preferred prediction error criteria. These error prediction criteria apply appropriate smoothness to model terms to minimize the GCV or GACV score, and, thereby, maximize the performance of the models. GAM quantifies complex species–environment relationships and reliable to fit local species occurrences (Aguirre‐Gutiérrez et al., 2013). On the other hand, the structural equation model (SEM), a linear regression technique is often used to validate the output of another model such as GAM (Wu, Wurst, & Zhang, 2016). It establishes causal relationships between multiple predictors and response variables within a single framework (Grace, 2006).

We used GAM to find relationships between species richness and the eight selected variables both at independent and at cumulative levels with/without interaction(s). We fitted GAM with a Poisson error distribution with a “log” link function using the package “mgcv” in RStudio (Wood, 2016). We fitted the c*ubic spline* (s) smoother to variables without interactive terms and the *tensor product* (te) smoother to interactive terms. We selected GCV (GCV.Cp) as prediction error criterion, which automatically selects models with the least error and improves the explanatory ability of models. The significance of each predictor was quantified using the *deviance* (%) [calculated as (null deviance—residual deviance/null deviance) × 100]. The data were split into four groups, and then, 1 group was held out and the model fit to the other three groups combined, that model then test on the held‐out group to get the performance. This process was repeated, with each of the four groups acting as the held‐out group, and overall *R* ^2^ taken as an average across all held‐out groups. We used the package *“*lavaan” in RStudio for SEM to examine the performance of the GAM predictions (Rosseel, 2012). We derived structural equations for a different set of variables and categories and plotted them in figures wherein different colors were assigned to represent positive/negative correlation, and the thickness of arrows explained the impact of independent variables on the dependent variable (Figure 3). The physiography, climate, and disturbance variables were grouped, and a separate SEM was fitted for each group. Further, we plotted the response curves of four climate variables to analyze the multidimensional regression through a two‐dimensional representation (Figure 4).

## 3 RESULTS

The two states of Western Himalaya have 1,279 species (sp.) in 645 genera and 176 families. These include 815 herbs, 213 shrubs, 190 trees, and 61 lianas, with species with missing information excluded (see Appendix S2). The family Poaceae is the largest family, with 141 species. The other dominant families are Asteraceae (119 sp.), Papilionaceae (70 sp.), Rosaceae (56 sp.), Polygonaceae (43 sp.), and Lamiaceae (40 sp.). *Polygonum* (21 sp.) is the most speciose genus. *Potentilla* (17 sp.), *Carex* (14 sp.), *Impatiens* (14 sp.), *Dryopteris* (13 sp.), and *Artemisia* (13 sp.) are other prominent genera. Moran's I value for the 1,178 spatial location points of the 738 sample plots was 0.39 at the *p* < .001 level of significance.

**Figure 2:**
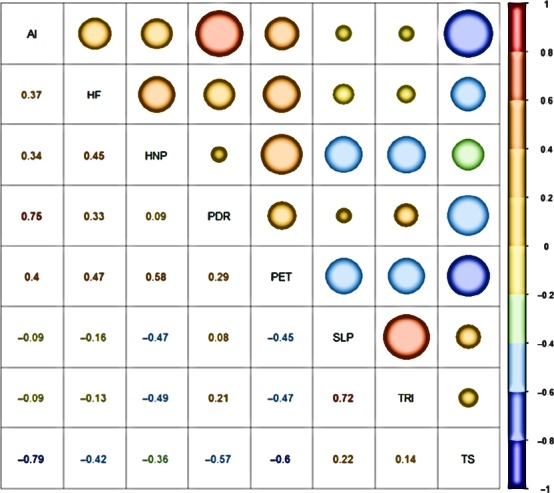
Correlation plot

The first three PC axes of the climate variables explained 61.77%, 17.95%, and 9.08% variance, respectively. They cumulatively explained >91.8% variance, and the percentage absolute weight of each variable varies between 11.39% and 20.49%. We found at least three sets of collinear variables (Appendix S3). One set of variables was led by potential evapotranspiration (PET) with the maximum percentage absolute weight (20.48%), which showed a very strong correlation (*R* > 0.9) with the mean annual temperature, temperature of the coldest month, temperature of the coldest quarter, temperature of the driest quarter, temperature of the wettest month, temperature of the wettest quarter, and temperature of the warmest quarter, and a strong correlation (*R* > 0.8) with precipitation seasonality and temperature of the wettest quarter (Appendix S4). The second set of variables was led by the aridity index, which showed a strong correlation (*R* > 0.8) with the mean annual precipitation, precipitation of the wettest month, precipitation of the wettest quarter, precipitation of the warmest quarter. Temperature seasonality led the next set of variables, which exhibited a very strong correlation with the temperature annual range and a strong correlation with the annual precipitation, precipitation of the warmest month, precipitation of the warmest quarter, precipitation of the wettest quarter, temperature of the coldest month, and temperature of the coldest quarter. The precipitation of the driest quarter was the fourth climatic variable selected after elimination of collinear variables (Appendix S4). The first three axes of the physiographic variables explained 48.8%, 26.2%, and 16.1% variance, respectively. Together they explained >91% variance, and the percentage absolute weight of these variables lies between 36.48% and 44.77%. The mean elevation is shown to have the maximum percentage absolute weight (44.77%), followed by slope (41.35%), terrain ruggedness index (38.41%), and aspect (36.48%). None of these physiographic variables were strongly correlated with each other (Appendix S4). However, the mean elevation was not considered for its strong correlation with selected climatic variables (Figure S1; Appendix S4). The percentage absolute weights of slope and terrain ruggedness index were next to those of the mean elevation. They showed no strong correlation with the selected variables of other categories of variables and, therefore, selected for modeling. The eight least‐correlated variables two each from energy, water, physiography, and disturbance: aridity index, human appropriation of net primary productivity, global human footprint, precipitation of the driest quarter, PET, slope, terrain ruggedness index, and temperature seasonality were finally selected for modeling (Figure 2).

The study area was found to have a mean aridity of 0.13. The dryness of the northwest is greater than that of the southeast of the region. The mean precipitation in the driest quarter was 101.8 mm. The central and northwestern regions get the maximum precipitation during this quarter. The mean PET was 994 mm. It was found to decrease along the elevation gradient monotonously. The mean human appropriation of net primary productivity was well below average (24.73 PgC/year) except in the southwestern parts of the study area, where the population density is high. On the other hand, the mean global human footprint was medium to above average across the study area (31.76/ha). The minimum and the maximum elevations were 207 m and 5,468 m, respectively. The mean terrain ruggedness index was high, at 656.86, whereas the mean slope was low, at 11.2°. Both showed similar spatial variations along the elevation gradient, that is, high ruggedness and slope were greater at higher elevations. All the selected variables were within the accepted level of skewness (Appendix S4).

The multimodel inference produced 45 combinations of variables (i.e., models; Tables 1 and 2). Fifteen noninteractive variable combinations were found to be significant for species richness pattern (Table 1). Except in a few cases, each variable within the models was significant at *p* < .001. Although some variables were insignificant, the *p*‐values of all the baseline SEM models were significant (*p* < .001). In GAM, no variable was found to be insignificant, and all cross‐validation results were significant at the *p* < .05 level at least (Table 1). Both approaches diagnosed the independent and cumulative effects of the energy variables to be significant for the species richness pattern. The temperature seasonality was the best predictor of species richness, and PET was the second‐best predictor. The best GAM model explained 43% variance by cross‐validation, whereas the best SEM model described 38% variance of species richness. SEM predicted the cumulative effects of water and disturbance at par with climate, that is, at 38%.

**Table 1:** Regression statistics for variable combinations without interactive terms using both structural equation model (SEM) and generalized additive model (GAM); results of GAM were computed using repeated cross‐validation; except in few cases, the *p* value of each variable within the models was significant at *p* < .001. Both approaches supported the same conclusion that energy (temperature seasonality and potential evapotranspiration) was the best predictor of species richness, and the mutual influence of common predictors is more significant than their cumulative effects

Model	Formula	SEM_R^2^	GAM_R^2^	% Deviance explained
M13	SR~s(PET) + s(TS)	0.30(0.39)	0.45	48.5
M22	SR~s(**AI**) + s(PDR) + s(PET) + s(TS)	0.38(nr)	0.43	54.8
M24	SR~s(**HF**) + s(HNP) + s(**AI**) + s(PDR) + s(PET) + s(TS)	0.31(0.37)	0.42	58.6
M20	SR~s(**HF)** + s(HNP) + s(**AI**) + s(PDR)	0.38(0.41)	0.41	53.9
M21	SR~s(HF) + s(HNP) + s(PET) + s(TS)	0.32(0.36)	0.41	52.6
M8	SR~s(TS)	0.27	0.40	44.8
M19	SR~s(**SLP**) + s(TRI) + s(PET) + s(TS)	0.35(0.38)	0.40	52.2
M18	SR~s(**SLP**) + s(TRI) + s(AI) + s(PDR)	0.31(nr)	0.38	50.2
M11	SR~s(AI) + s(PDR)	0.29(0.34)	0.37	45.6
M5	SR~s(AI)	0.25	0.35	41.2
M7	SR~s(PET)	0.26	0.34	35.6
M6	SR~s(PDR)	0.21	0.30	34.8
M10	SR~s(HF) + s(HNP)	0.18(0.29)	0.23	26.0
M3	SR~s(HF)	0.12	0.17	19.2
M4	SR~s(HNP)	0.14	0.17	16.9

SR, Species richness; PET, potential evapotranspiration; AI, aridity index; PDR, precipitation of the driest quarter; TS, temperature seasonality; SLP, slope; TRI, terrain ruggedness index; HNP, human appropriation of net primary productivity; HF, global human footprint.

In SEM, some variables predicted insignificant (*p* > .05) are highlighted. HF and PET of M21 were significant at *p* < .05 and *p* < .01, respectively, for the same model; in GAM, HF of M20, M21, and M24 models was significant at *p* < .05, *p* < .05, and *p* < .01, respectively. “nr” indicates “no results”; The *R* ^2^ values the proportion of variance explained in the held‐out group in the cross‐validation.

**Table 2:** Generalized additive model‐derived regression statistics for models with interactive terms; models M28–M45 are variables combinations with interactive terms. Cubic spline smoother(s) fitted to noninteractive terms and tensor product (te) smoother to interaction terms; except in few cases, the *p* value of each variable within the models was significant at *p* < .001

Model	Formula	% Deviance explained
M45	SR~s(HNP)* + s(**HF**) + te(HNP,HF) + s(SLP) + s(**TRI**) + te(SLP, TRI) + s(AI) + s(**PDR**) + te(AI,PDR) + s(PET) + s(TS) + te(PET,TS)	67.4
M43	SR~s(SLP) + s(**TRI**) + te(SLP, TRI) + s(AI) + s(**PDR**) + te(AI,PDR) + s(**PET**) + s(TS) + te(PET,TS)	63.7
M44	SR~s(HNP) + s(HF)*** + te(**HF,HNP**)*** + s(AI) + s(PDR)*** + te(AI,PDR) + s(**PET**) + s(TS) + te(PET,TS)	63.3
M36	SR~s(AI) + s(PDR) + te(AI,PDR) + s(PET)* + s(TS) + te(PET,TS)	62.1
M41	SR~s(HF) + s(HNP)** + te(HF,HNP) + s(**PET**) + s(TS) + te(PET,TS)	60.1
M38	SR~s(AI) + s(PDR)*** + s(PET) + s(TS) + te(AI,TS) + te(PDR,PET)	60.0
M37	SR~s(AI) + s(PDR) + s(PET) + s(TS) + te(AI,PET) + te(PDR,TS)	59.0
M39	SR~s(SLP) + s(**TRI**) + te(SLP, TRI) + s(**PET**) + s(TS) + te(PET,TS)	58.9
M42	SR~s(**HF**)*** + s(HNP) + te(HF,HNP)** + s(AI) + s(PDR) + te(AI,PDR)	56.2
M35	SR~s(PET) + s(TS) + te(PET,TS)	54.7
M40	SR~s(SLP) + s(TRI)* + te(SLP, TRI) + s(AI) + s(PDR)** + te(AI,PDR)	52.8
M34	SR~s(AI)*** + s(PDR) + te(AI,PDR)	49.7
M31	SR~te(PET,TS)	47.5
M30	SR~te(AI,PDR)	44.0
M33	SR~s(HNP) + s(HF) + te(HNP,HF)	32.4
M29	SR~te(HF,HNP)	27.1
M32	SR~s(SLP) + s(TRI) + te(SLP,TRI)***	10.6
M28	SR~te(SLP,TRI)	7.8

Variables highlighted are not statistically significant. “**,” “*,”and “***” indicate significance at *p* < .01, *p* < .05, and *p* < .1, respectively.

The best two SEM model predictions are as follows:

(1) Water+Disturbance

$$\begin{array}{cc} \text{SR}\;=\; & 14.690\;+\;0.148\;*\;\text{HF}-0.271\;*\;\\ & \text{HNP}\;+\;0.479\;*\;\text{AI}\;+\;0.399\;*\;\text{PDR} \end{array}$$

(2) Climate(Water+Energy)

$$\begin{array}{cc} \text{SR}\;=\; & 12.194\;+\;0.112\;*\;\text{AI}\;+\;2.796\;*\;\text{PDR}\;+\;\\ & 2.849\;*\;\text{PET}-1.704\;*\;\text{TS} \end{array}$$

AI = Aridity index; HF = human footprint; HNP = human appropriation of net primary productivity; PDR = precipitation of driest quarter; PET = potential evapotranspiration; TS = temperature seasonality.

Interestingly, the percentage deviance explained by each GAM model was not proportionate with the cross‐validation results. For example, the most complex combination of climate and disturbance which described the maximum deviance, at 58.6%, was the second‐best model. The SEM model predictions showed better correlation with the deviance explained by GAM. Unlike GAM cross‐validation results, the best SEM model explained the maximum deviance.

**Figure 3:**
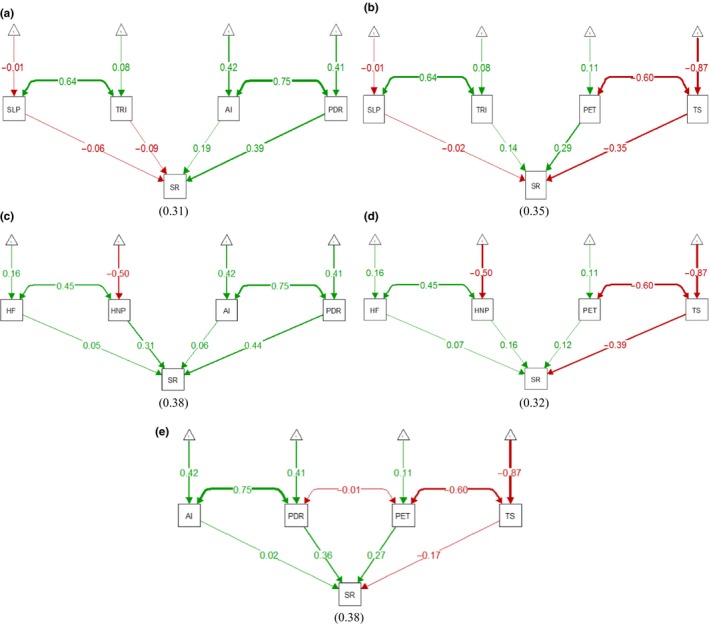
(a) Effects of physiography and water on species richness. (b) Effects of physiography and energy on species richness. (c) Effects of disturbance and water on species richness. (d) Effects of disturbance and energy on species richness. (e) Effects of energy and water on species richness

**Figure 4:**
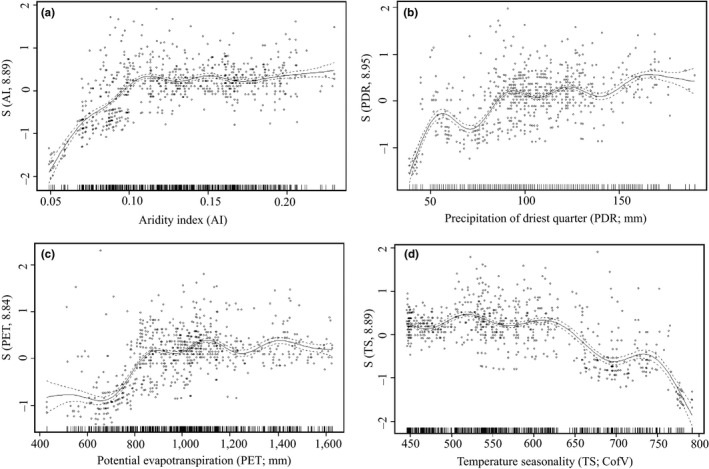
Curves showing species response function with (a, b) water and (c, d) energy variables

Structural equation model predicted negative relationships between the aridity index and temperature seasonality with species richness. The precipitation of the driest quarter and PET were found to have positive correlations with species richness. The global human footprint and human appropriation of net primary productivity showed weak positive and weak negative correlations with species richness. Slope and terrain ruggedness index were found to have weak negative and weak positive correlations with species richness, respectively (Figure 3). The response curves of GAM models showed trends similar to those of the SEM predictions. The piecewise polynomial curves were closely fitted with low degrees of freedom (7—9), that is, they neither straight nor wiggled. The aridity index had a linear and upward trend, but it became more or less straight after an index value of 0.10. Its variation from the mean zero level was found to be insignificant (Figure 4a). In contrast, the precipitation of the driest quarter exhibited an irregular curve pattern and differed greatly from the mean zero level. It showed a general positive trend with species richness (Figure 4b). PET showed a greater variation from the mean zero level and a positive relationship with species richness (Figure 4c). Temperature seasonality showed a complex curve pattern and was negatively correlated with species richness after approximately 625 CofV from the mean zero level (Figure 4d).

Structural equation model showed better goodness of fit with interactions. The latent variables (groups of common predictors) were found to influence the species richness better compared with the combined effect of independent variables of the same groups (Table 1). The best model involving latent variables described 41% variance, the maximum predicted by any SEM model. The influences of physiography and disturbance with groups of common climatic predictors fitted better than their influences with separate groups of water and energy predictors (Figure 3). GAM exhibited similar relationships, that is, enhanced performance with inclusion of interactive terms in the models. The performance of models improved with complexities. The full model with a combination of all selected variables described the maximum deviance of 65.7% (Table 2). With climate, physiography showed greater significance than did disturbance. The deviance explained by common predictors of any two sets of variables with inclusion of interactive terms was between 52.8% and 62.1%, and the base climate model explained the deviance to the extent of 62.1%. In general, the inclusion of interactive terms of common predictors with noninteractive terms enhanced the percentage of the explained deviance. With inclusion of interactive terms, the deviance explained by energy was 54.7%, a 5% greater value than the deviance explained by water. Comparatively, the interactions between the disturbance variables explained more deviance than did the physiographic variables (Table 2).

## 4 DISCUSSION

The dominant presence of herbaceous plant families, specifically Poaceae, Asteraceae, and Rosaceae, indicates the prevalence of grassland vegetation in the area. Similar familial relationships have also been reported by Rashid et al. (2013) in biodiversity‐rich areas of the northern Himalaya with predominantly grassland vegetation.

Analyzing relationships of plant species richness with climate, disturbance, and physiography using both GAM and SEM allows us to compare the relative importance of abiotic predictors in describing the variations in the dry and cold Western Himalaya. Both models indicate a significant contribution of climate to the species richness pattern. Our results corroborate the findings of Chitale (2014), who reported that Himalayan ecology is broadly defined by climate. Several studies describe strong relationships between climate and species richness (Clark et al., 1999; Curie et al., 2004; Hawkins et al., 2003; Ricklefs, 1987; Wang et al., 2007). Our results show the key roles of the water–energy dynamics in climatic control in explaining the capacity of species richness (O'Brien, 1993; O'Brien, Whittaker, & Field, 1998). The energy variables, that is, temperature seasonality and PET, are the most significant determinants of species richness patterns. The prevailing cold and dry conditions of the area might have led to the strong biotic dependency on energy. Many ecologists agree that energy has greater control in cold climates (Francis & Currie, 2003; Kreft & Jetz, 2007). The energy hypothesis proposes that greater availability of energy in a usable form enhances the species richness (Turner et al., 1987). The water variables show a strong association with species richness, but with a relatively lower magnitude compared with the energy variables. Water is an essential solvent for richness patterns in the tropics, subtropics, and warm temperate zones (Hawkins et al., 2003). However, our results contradict with the findings of Hawkins et al. (2006), who demonstrated a stronger influence of precipitation compared with energy in high to mid‐latitudes. The geographic position and evolutionary history of the area might have shaped this water and energy transitions with reference to species richness (Xu et al., 2016). The species–energy affinities may be associated with Himalaya's youthful physiography and unstable geology (Mani, 1974). Although water and energy contribute differently to the species richness pattern, their synergy defines the mechanism by which the contemporary climate affects the species richness pattern. Water stress negatively affects species richness in spite of availability of sufficient energy, and with optimum water availability, plants would exploit the maximum photon flux essential for their physiological activities.

Water–energy variables and their interactions have primacy over anthropogenic disturbance and physiography. Independently, energy and water variables have primacy over nonclimatic variables. The temperature seasonality is the greatest contributor, which indicates a significant influence of seasonal variation in temperature on the species richness. This variable exhibits complex, but negative correlations with species richness. This explains why the ecological stability of the region is balanced by fluctuations in temperature, and a global rise in temperature would be significant in controlling plant richness in future climates. PET is the second‐best predictor of plant richness. It is a crucial factor in the Himalayan region (Chitale, 2014). PET has been reported to be the best predictor of the species richness pattern (O'Brien, 1993). A low level of PET shows positive relationships with the species richness. However, PET saturates at above 900 mm with no significant variations indicating the indirect influence of elevation on the species richness pattern. The monotonous decrease in PET along elevation gradient probably explains this conjecture. The aridity index is the best‐contributing water variable, which explains the impact of water stress on the species richness. This index was a crucial factor in an arid climate (Li et al., 2013). To a certain extent, dryness may facilitate species richness, but an index value >0.10 is probably counterproductive for plant richness. In general, a positive relation between the precipitation of the driest quarter and species richness is predicted. It is likely that species in places of high mean dryness face greater stress due to water deficiency during the driest quarter. In contrast, dry northwestern parts of the study area get the maximum precipitation during the driest quarter, which might have reduced the adverse effect of dryness on species richness. Anthropogenic disturbance has a key role in determining the species richness pattern of Western Himalaya (Gupta, 1978; Kharkwal, 2009; Negi et al., 2012; Shrestha et al., 2012). The two disturbance variables could explain the species richness almost equivalently. However, human appropriation of net primary productivity has a positive correlation with species richness, whereas global human footprint has a negative correlation. This indicates that some level of disturbance is favorable for increasing species richness. The negative impact of global human footprint indicates that frequency of human interference may adversely affect the species diversity of the region. Although the correlation between global human footprint and species richness is weak, it is likely to have a significant influence in the regions of high population density. The mean human appropriation of net primary productivity in the southwestern region and the mean global human footprint at flat elevated surfaces indicate anthropogenic disturbance in high‐populated areas. The areal proximity and dominant grassland vegetation might have increased the human impact of these regions. Sharma et al. (2009) described the positive correlation between fuel wood collection and forest dependence on forests of villagers to their poor socio‐ecological status.

We found physiography to be a weaker predictor of species richness compared with disturbance. This contradicts the finding of Chitale (2014) that physiography is a better predictor compared with anthropogenic disturbance in the Himalaya. The selection of variables might be the most important reason for this difference. Exclusion of elevation from the list of selected variables may be the most plausible reason for the disparities. The very strong correlation between the mean elevation and PET describes the indirect influence of elevation on species richness. Their negative relationship is indicative of decreasing species richness along the elevation gradient. An indirect association of elevation on species richness by controlling the climate and soil has been reported (Day & Monk, 1974). Elevation has the strong influence on the vegetation in most mountain ecosystems (Zhang et al., 2009). The enhancement of the performance of the model upon addition of physiographic variables to climatic predictors probably explains the influence of physical heterogeneity on species’ niche differentiation (Whittaker et al., 1973). Slope is found to be weak predictor in describing the plant richness pattern. This probably explains the influence of the dominant grassland vegetation of the study area, which set the microclimate for herbaceous plants. It differs in describing positive relationships between slope and tree species (Bianchini et al., 2010). Slope is reported to be a key factor in determining the vegetation at a local scale (Kirkby et al., 1990), but it may not be a significant factor at the landscape or regional scale.

The explanatory power of the models improved significantly with inclusion of interactive terms (Table 2). Interactions between common predictors showed proportionate variations relative to base models and with greater efficiency, that is, energy>water>disturbance>physiography. This indicates that mutual influences of common predictors on the species richness are more significant than their independent effects. Climatic variables show a greater synergy with each compared with nonclimatic variables. The SEM results substantiate GAM predictions. The mutual influences of common predictors (i.e., latent variables) are more significant than the cumulative effects of independent predictors. This indicates that multiple parameters act synergistically to shape the species richness pattern. The competence of the linear structural model is equivalent to that of the nonlinear model, but with low efficiency. It describes that species and environment relationships are more likely to be nonlinear than to have linear fit. In general, both models identified climate has the primacy in determining the species richness pattern. The energy variables, that is, temperature seasonality and PET, are the most significant determinants of the species richness pattern. The interactions between climatic variables are more critical than those of nonclimatic variables. The overall explanatory ability improves with the complexity of the model, and a full model with all variables and their corresponding interactive terms could explain a maximum deviance of 67.4%.

## 5 CONCLUDING REMARKS

This study used a newly available national biodiversity database for India to explore species–environment relationships in two states in Western Himalaya on the basis of environmental variables and human impact. With GAM and SEM models including climatic, physiographic, and anthropogenic variables, energy variables, that is, temperature seasonality and PET, were most significant in explaining the species richness pattern. Water–energy variables and their interactions had primacy over anthropogenic disturbance and physiography. Disturbance is a critical factor in the southwestern region and places of low elevations with large populations. Physiography is less significant compared with disturbance. The exclusion of elevation from the list of selected variables may be significant in this low performance of the physiographic variables with respect to species richness. Further investigations may improve the findings if elevation is included in the list of predictors. Additionally, the better correlation of species richness with energy than with water needs to be verified in different climatic regions. Although this study focuses on disentangling the effects of abiotic predictors on species richness, the relationships between the biotic factors, that is, dispersion, competition, and isolation, are likely to improve the reliability and performance of the models. Nevertheless, the present study provides better insights into ecologists and planners dealing with plant distribution patterns vis‐à‐vis climate change projections.

## AUTHOR CONTRIBUTIONS

Conceptualization: MDB. Formal analysis: RMP. Investigation: MDB RMP. Methodology: RMP. Software: RMP. Supervision: MDB. Writing—Original draft: RMP. Reviewing: MDB RMP PSR CB. Permission to use species data: PSR.
