# Supplementary material for: Energy determines broad pattern of plant distribution in Western Himalaya
Source: Ecol Evol. 2017 Nov 10;7(24):10850–60. doi: 10.1002/ece3.3569 (PMC5743696; doi:10.1002/ece3.3569)
Supplement: Supplementary file 1 [file ECE3-7-10850-s001.pptx]

## Slide 1
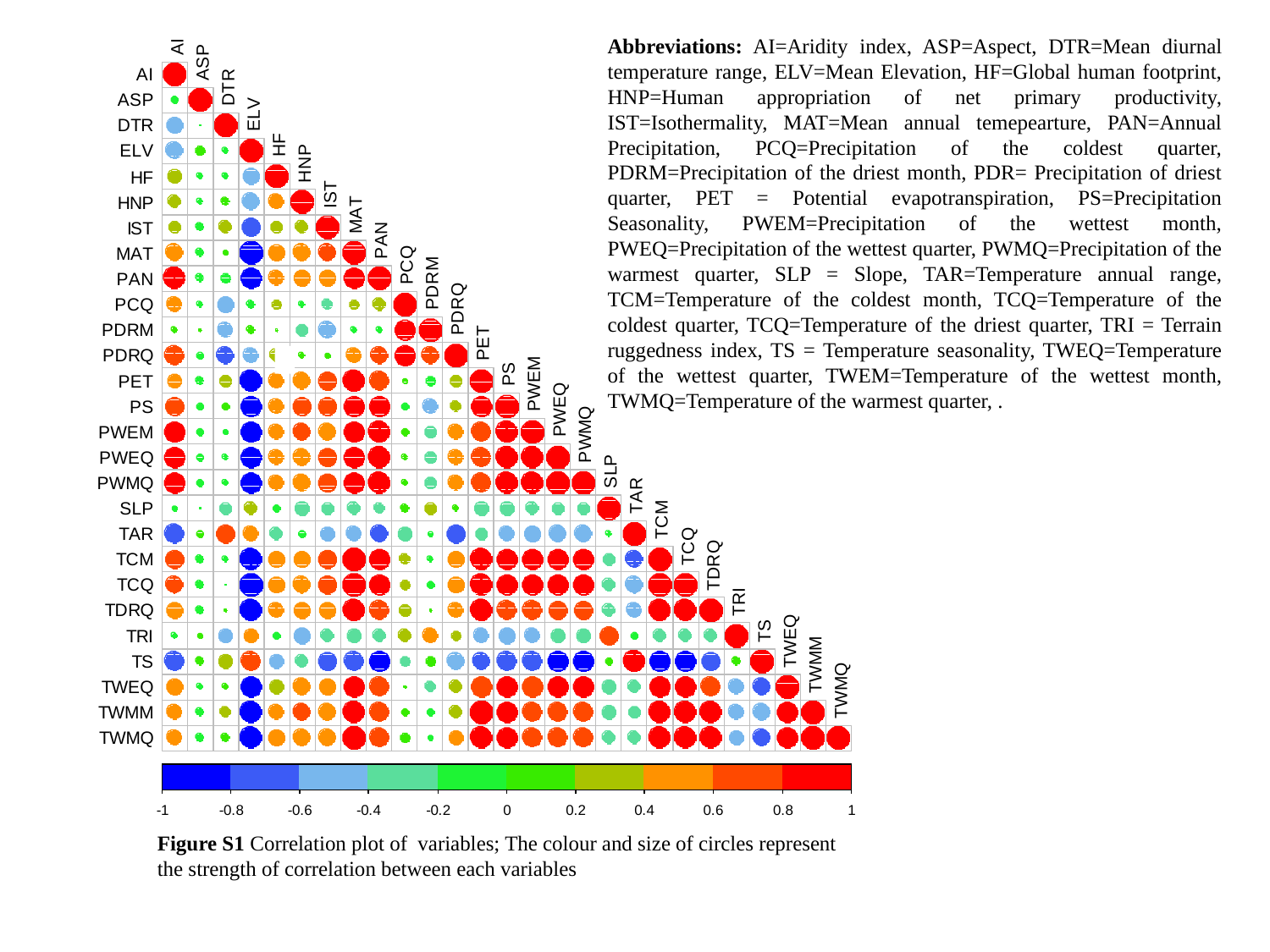

Abbreviations: AI=Aridity index, ASP=Aspect, DTR=Mean diurnal temperature range, ELV=Mean Elevation, HF=Global human footprint, HNP=Human appropriation of net primary productivity, IST=Isothermality, MAT=Mean annual temepearture, PAN=Annual Precipitation, PCQ=Precipitation of the coldest quarter, PDRM=Precipitation of the driest month, PDR= Precipitation of driest quarter, PET = Potential evapotranspiration, PS=Precipitation Seasonality, PWEM=Precipitation of the wettest month, PWEQ=Precipitation of the wettest quarter, PWMQ=Precipitation of the warmest quarter, SLP = Slope, TAR=Temperature annual range, TCM=Temperature of the coldest month, TCQ=Temperature of the coldest quarter, TCQ=Temperature of the driest quarter, TRI = Terrain ruggedness index, TS = Temperature seasonality, TWEQ=Temperature of the wettest quarter, TWEM=Temperature of the wettest month, TWMQ=Temperature of the warmest quarter, .
Figure S1 Correlation plot of variables; The colour and size of circles represent the strength of correlation between each variables
