# Supplementary material for: Energy determines broad pattern of plant distribution in Western Himalaya
Source: Ecol Evol. 2017 Nov 10;7(24):10850–60. doi: 10.1002/ece3.3569 (PMC5743696; doi:10.1002/ece3.3569)
Supplement: Supplementary file 2 [file ECE3-7-10850-s002.pptx]

## Slide 1
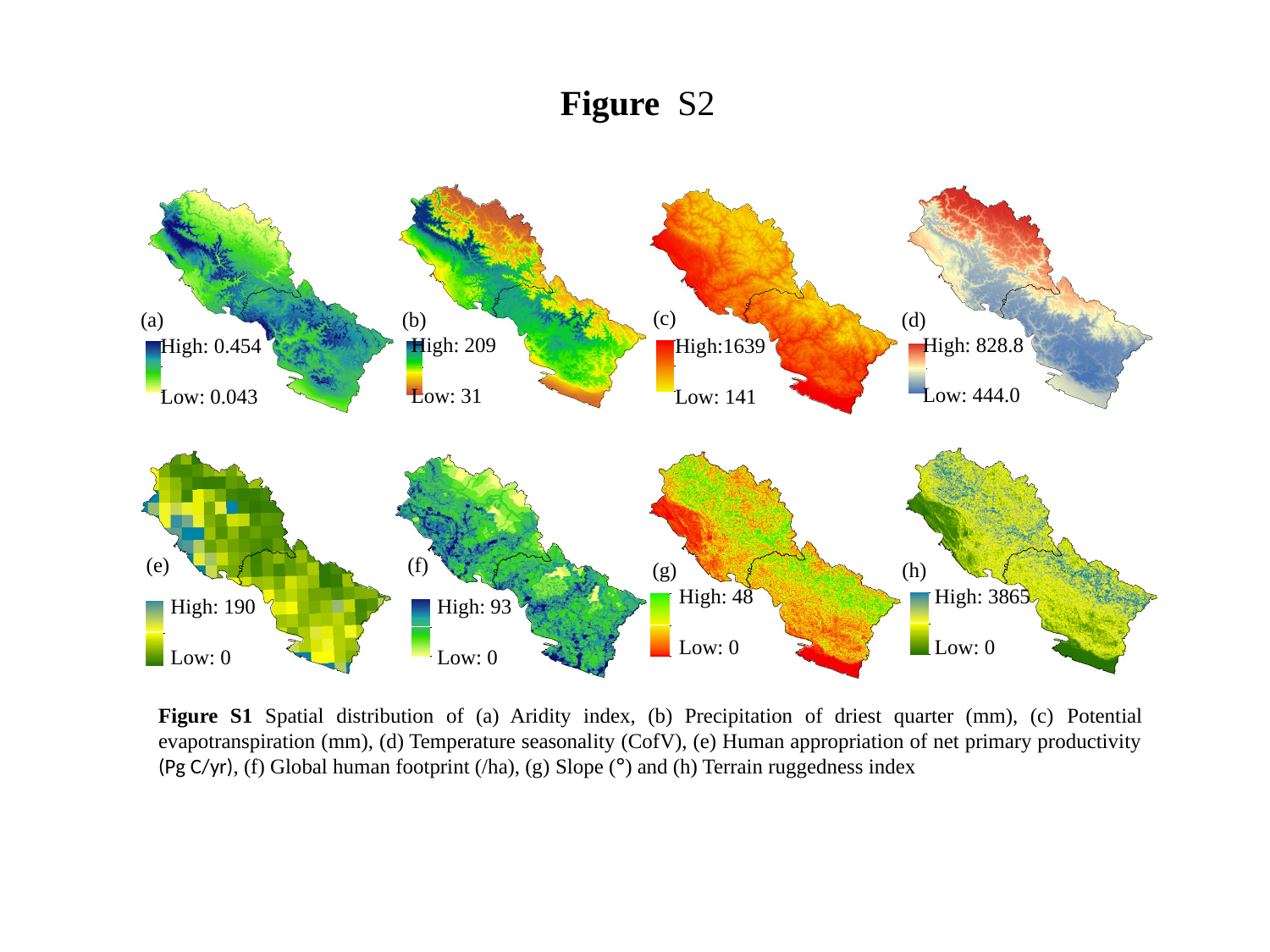

Figure S2
High: 209
Low: 31
(b)
High: 828.8
Low: 444.0
(d)
High: 0.454
Low: 0.043
(a)
High:1639
Low: 141
(c)
High: 190
Low: 0
High: 3865
Low: 0
High: 93
Low: 0
High: 48
Low: 0
(f)
(e)
(h)
(g)
Figure S1 Spatial distribution of (a) Aridity index, (b) Precipitation of driest quarter (mm), (c) Potential evapotranspiration (mm), (d) Temperature seasonality (CofV), (e) Human appropriation of net primary productivity (Pg C/yr), (f) Global human footprint (/ha), (g) Slope (°) and (h) Terrain ruggedness index
