# Supplementary material for: Energy determines broad pattern of plant distribution in Western Himalaya
Source: Ecol Evol. 2017 Nov 10;7(24):10850–60. doi: 10.1002/ece3.3569 (PMC5743696; doi:10.1002/ece3.3569)
Supplement: Supplementary file 3 [file ECE3-7-10850-s003.docx]

| Variable Type | Data Layers | Time Period | Sources |
| --- | --- | --- | --- |
| Physiographic | Mean Elevation (ELEV) | 2010 | USGS |
|  | Slope (SLP) |  | https://lta.cr.usgs.gov/ |
|  | Aspect (ASP) |  | GMTED2010 |
|  | Terrain Ruggedness Index (TRI) |  |  |
| Climate | Annual Mean Temperature (MAT) | 1950−2000 |  |
|  | Mean Diurnal Temperature Range (DTR) |  |  |
|  | Isothermality (IST) |  |  |
|  | Temperature Seasonality (TS) |  |  |
|  | Maximum Temperature of the Warmest Month (TWMM) |  |  |
|  | Minimum Temperature of the Coldest Month (TCM) |  |  |
|  | Temperature Annual Range (TAR) |  |  |
|  | Mean Temperature of the Wettest Quarter (TWEQ) |  |  |
|  | Mean Temperature of the Driest Quarter (TDRQ) |  |  |
|  | Mean Temperature of the Warmest Quarter (TWMQ) |  |  |
|  | Mean Temperature of the Coldest Quarter (TCQ) |  | WORLDCLIM v1.3 |
|  | Annual Precipitation (PAN) |  | http://www.worldclim.org/ |
|  | Precipitation of the Wettest Month (PWEM) |  |  |
|  | Precipitation of the Driest Month (PDRM) |  |  |
|  | Precipitation Seasonality (PS) |  |  |
|  | Precipitation of the Wettest Quarter (PWEQ) |  |  |
|  | Precipitation of the Driest Quarter (PDR) |  |  |
|  | Precipitation of the Warmest Quarter (PWMQ) |  |  |
|  | Precipitation of Coldest Quarter (PCQ) |  |  |
|  | Potential Evapotranspiration (PET) |  | CGIAR-CSI |
|  | Annual aridity index (AI) |  | http://www.cgiar-csi.org/ |
| Disturbance | Human Appropriation of Net Primary Productivity | 1995, 1995-2004 | CIESIN-SEDAC  http://sedac.ciesin.columbia.edu/ |
|  | Global Human Footprint |  |  |

**Appendix S1**

Data Types and Sources
