# Supplementary material for: Energy determines broad pattern of plant distribution in Western Himalaya
Source: Ecol Evol. 2017 Nov 10;7(24):10850–60. doi: 10.1002/ece3.3569 (PMC5743696; doi:10.1002/ece3.3569)
Supplement: Supplementary file 5 [file ECE3-7-10850-s005.docx]

| Variables | Univariate Analysis | | | Principal Component Analysis | | | |
| --- | --- | --- | --- | --- | --- | --- | --- |
| Climate | Mean | Std. Dev. | Skew-ness | PC1 (64.7 %) | PC2 (17.9 %) | PC3 (9.1 %) | % Total Absolute Weight |
| DTR | 9.65 | 1.09 | 0.41 | 0.019 | 0.447 | -0.233 | 11.400 |
| PDRM | 15.09 | 6.37 | 0.16 | 0.043 | -0.407 | -0.406 | 13.753 |
| PCQ | 186.95 | 67.32 | 0.66 | -0.056 | -0.438 | -0.277 | 13.984 |
| **TWEQ** | 13.59 | 9.27 | -0.55 | -0.231 | 0.095 | -0.047 | 17.093 |
| IST | 35.63 | 3.24 | 0.50 | -0.183 | 0.224 | 0.162 | 17.356 |
| *PDR* | 101.80 | 30.98 | 0.02 | -0.146 | -0.408 | -0.100 | 17.700 |
| PWEM | 311.67 | 203.01 | 0.24 | -0.254 | 0.026 | 0.122 | 18.022 |
| **TCM*** | -2.93 | 8.00 | -0.66 | -0.264 | -0.006 | -0.100 | 18.149 |
| **TDRQ** | 7.52 | 7.11 | -0.18 | -0.244 | 0.013 | -0.254 | 18.351 |
| PWEQ* | 474.07 | 278.09 | 0.02 | -0.253 | 0.014 | 0.192 | 18.370 |
| PWMQ* | 474.07 | 278.09 | 0.02 | -0.253 | 0.014 | 0.192 | 18.370 |
| TAR* | 26.82 | 3.16 | 0.37 | 0.168 | 0.267 | -0.311 | 18.508 |
| **TCQ*** | 3.03 | 7.91 | -0.51 | -0.264 | 0.022 | -0.120 | 18.595 |
| **PAN*** | 1290.73 | 574.58 | -0.01 | -0.256 | -0.079 | 0.086 | 18.801 |
| **MAT** | 11.11 | 7.08 | -0.31 | -0.259 | 0.042 | -0.183 | 19.208 |
| *TS** | 581.46 | 100.78 | 0.45 | 0.232 | 0.106 | -0.260 | 19.258 |
| **TWMQ*** | 17.85 | 6.00 | 0.08 | -0.247 | 0.074 | -0.270 | 19.753 |
| **PS** | 74.52 | 31.12 | 0.13 | -0.248 | 0.143 | 0.128 | 19.795 |
| *AI* | 0.13 | 0.04 | 0.19 | -0.204 | -0.245 | 0.254 | 19.904 |
| **TWMM*** | 23.89 | 6.54 | 0.38 | -0.242 | 0.121 | -0.272 | 20.321 |
| ***PET*** | 994.03 | 247.22 | 0.44 | -0.241 | 0.153 | -0.236 | 20.482 |
| Physiographic | Mean | Std. Dev. | Skew-ness | PC1 (48.8 %) | PC2 (26.2 %) | PC3 (16.1 %) | % Absolute Weight Total |
| ASP | 187.573 | 107.39 | -0.16 | 0.128 | 0.911 | -0.389 | 36.383 |
| ELV | 2527.78 | 1213.4 | 0.242 | 0.500 | 0.275 | 0.817 | 44.780 |
| *SLP* | 11.8123 | 7.0667 | 0.476 | 0.594 | -0.261 | -0.341 | 41.354 |
| *TRI* | 700.791 | 295.3 | -0.1 | 0.617 | -0.160 | -0.253 | 38.413 |

**Appendix S3** Univariate Statistics, Correlation and Principal Component (PC) loadings of climate and physiographic variables; Variance explained by each PC axis are given in parentheses; Percentage total of absolute weight of variables is the summation of product of % variance explained to PC loadings of each axis; Variables collinear with potential evapotranspiration, aridity index and temperature seasonality are represented with bold letters, underlines and star marks respectively; Selected variables are in Italics.

**Abbreviations:** AI=Aridity index, ASP=Aspect, DTR=Mean diurnal temperature range, ELV=Mean Elevation, IST=Isothermality, MAT=Mean annual temperature, PAN=Annual Precipitation, PCQ=Precipitation of the coldest quarter, PDRM=Precipitation of the driest month, PDR=Precipitation of driest quarter, PET=Potential evapotranspiration, PS=Precipitation Seasonality, PWEM=Precipitation of the wettest month, PWEQ=Precipitation of the wettest quarter, PWMQ=Precipitation of the warmest quarter, SLP =Slope, TAR=Temperature annual range, TCM=Temperature of the coldest month, TCQ=Temperature of the coldest quarter, TCQ=Temperature of the driest quarter, TRI = Terrain ruggedness index, TS = Temperature seasonality, TWEQ=Temperature of the wettest quarter, TWEM=Temperature of the wettest month, TWMQ=Temperature of the warmest quarter, .
